# Prolonged viral pneumonia and high mortality in COVID-19 patients on anti-CD20 monoclonal antibody therapy

**DOI:** 10.1007/s10096-024-04776-0

**Published:** 2024-02-15

**Authors:** Eeva Feuth, Valtteri Nieminen, Antti Palomäki, Juha Ranti, Marcus Sucksdorff, Taru Finnilä, Jarmo Oksi, Tytti Vuorinen, Thijs Feuth

**Affiliations:** 1 Department of Infectious Diseases, Turku University Hospital and University of Turku, Turku, Finland; 2 Department of Pulmonary Diseases and Clinical Allergology, Turku University Hospital and University of Turku, Turku, Finland; 3 Centre for Rheumatology and Clinical Immunology, and Department of Medicine, Turku University Hospital and University of Turku, Turku, Finland; 4 Department of Haematology, Turku University Hospital, Turku, Finland; 5 Turku PET Centre, and Division of Clinical Neurosciences, Turku University Hospital and University of Turku, Turku, Finland; 6 Department of Hospital Hygiene & Infection Control, Turku University Hospital, Turku, Finland; 7 Department of Clinical Microbiology, Turku University Hospital and Institute of Biomedicine, University of Turku, Turku, Finland

**Keywords:** COVID-19, SARS-CoV-2, Anti-CD20 mAb, Rituximab, Immunosuppression, Viral pneumonia

## Abstract

**Purpose:**

In clinical practice, we observed an apparent overrepresentation of COVID-19 patients on anti-CD20 monoclonal antibody therapy. The aim of this study was to characterize the clinical picture of COVID-19 in these patients.

**Methods:**

All adult patients from Turku University Hospital, Turku, Finland, with COVID-19 diagnosis and/or positive SARS-CoV-2 PCR test result up to March 2023, and with anti-CD20 therapy within 12 months before COVID-19 were included. Data was retrospectively obtained from electronic patient records.

**Results:**

Ninety-eight patients were identified. 44/93 patients (47.3%) were hospitalized due to COVID-19. Patients with demyelinating disorder (*n* = 20) were youngest (median age 36.5 years, interquartile range 33–45 years), had less comorbidities, and were least likely to be hospitalized (2/20; 10.0%) or die (*n* = 0). COVID-19 mortality was 13.3% in the whole group, with age and male sex as independent risk factors. Persistent symptoms were documented in 33/94 patients (35.1%) alive by day 30, in 21/89 patients (23.6%) after 60 days, and in 15/85 after 90 days (17.6%), mostly in patients with haematological malignancy or connective tissue disease. Prolonged symptoms after 60 days predisposed to persistent radiological findings (odds ratio 64.0; 95% confidence interval 6.3–711; *p* < 0.0001) and persistently positive PCR (odds ratio 45.5, 95% confidence interval 4.0–535; *p* < 0.0001). Several patients displayed rapid response to late antiviral therapy.

**Conclusion:**

Anti-CD20 monoclonal antibody therapy is associated with high COVID-19 mortality and with a phenotype consistent with prolonged viral pneumonia. Our study provides rationale for retesting of immunocompromised patients with prolonged COVID-19 symptoms and considering antiviral therapy.

**Supplementary Information:**

The online version contains supplementary material available at 10.1007/s10096-024-04776-0.

## Introduction

Vaccines against severe acute respiratory syndrome coronavirus 2 (SARS-CoV-2) infection provide good protection against severe COVID-19 for most people [1]. However, several groups of patients remain at risk for severe disease despite repeated vaccinations, including patients on certain immunosuppressive medications [2].

B-cells play a key role in adaptive immunity and lower B-cell levels in plasma are associated with poor outcome in COVID-19 [3]. Treatment with monoclonal antibodies (mAb) targeting the CD20 antigen on the surface of B-cells (anti-CD20 mAbs) are applied in several B-cell mediated diseases, including haematological disorders and malignancies, connective tissue diseases, rheumatoid arthritis, multiple sclerosis (MS), and vasculitis. Anti-CD20 mAb causes a depletion of B-cells and may induce hypogammaglobulinemia lasting up to 12 months [4]. During this time period, B-cells are not capable of producing antibodies against newly encountered pathogens and efficacy of vaccination may be severely diminished [5, 6]. Therefore, patients receiving anti-CD20 mAbs may be at risk for severe COVID-19 despite vaccination [7, 8]. Indeed, the plasma concentration of antibodies against SARS-CoV-2 in patients on anti-CD20 mAbs is low, which predisposes to poor outcome in COVID-19 [9, 10]. In comparison with general population, the risk of reinfection by the emerging variant strains might also be higher in patients on anti-CD20 mAb therapy due to diminished antibody response by B-cells and depleted memory B-cells [7]. Furthermore, a number of case reports have described a delayed clinical, microbiological, and/or radiological recovery after SARS-CoV-2 infection in patients on anti-CD20 mAb therapy [11–17]. Repeated courses of antivirals and/or convalescent plasma have been used in these patients with relapsing or persisting COVID-19 pneumonia [15–21].

In the third year of the COVID-19 pandemic, we noted an apparent overrepresentation of patients on anti-CD20 mAb therapy with severe COVID-19 in our hospital. Some of these patients displayed a phenotype consistent with prolonged viral pneumonia. Therefore, we designed this retrospective cohort study to explore the clinical picture of COVID-19 in patients on anti-CD20 mAb therapy.

## Materials and methods

### Study population

All adult patients (aged ≥ 18 years) in Turku University Hospital with COVID-19 between 1st of March 2020 and 31st of March 2023 and within 12 months after anti-CD20 mAb therapy were included in this study. COVID-19 patients were identified from the electronic hospital database by ICD-10 codes U007─U010 and/or by SARS-CoV-2 positive reverse transcription polymerase chain reaction (RT-PCR) test result. Of note, Clinical Microbiology laboratory in Turku University Hospital served as the central COVID-19 testing laboratory in the Hospital District of South-West Finland throughout the study period. Data was obtained only in relation to the first COVID-19 infection fitting these criteria.

### Data collection

Patients were divided into four groups according to primary indication for anti-CD20 mAb therapy: haematological malignancy (*n* = 40), connective tissue disease (CTD; *n* = 27), demyelinating disorder (*n* = 20), and “other” (*n* = 11). Patient characteristics at the time of the COVID-19 diagnosis, risk factors, and clinical characteristics were retrospectively collected from the electronic patient records. Vaccination status was obtained from the written notes in the patient records. SARS-CoV2 RT-PCR results and radiologic findings in chest computed tomography (CT) scans after day 30/60/90 were gathered retrospectively from patient records. All data were entered directly in the study database using REDcap (Vanderbilt University).

### Statistical analyses

We used descriptive statistics to characterize patients and compare groups. Categorical variables were displayed as numbers and percentages, while medians and quartiles (lower (Q1) and upper (Q3)) were used for continuous variables. Chi-square test was used to compare categorical variables between groups. Mann–Whitney U test was used to compare continuous or ordinal variables between two groups and Kruskal–Wallis for more than two groups, followed by Mann–Whitney U test to test differences between individual groups. Kaplan–Meier curves were applied for survival. Statistical tests were performed using IBM SPSS Statistics 27 for Windows.

## Results

### Patient characteristics

A total of 98 adult patients on anti-CD20 mAb therapy were included in this study. The first patient in our study had symptoms starting at 25th of December 2020. One had symptoms starting in the year 2020, 11 in 2021, 81 in 2022, and 5 in 2023.

Fifty-seven of 98 patients (58.2%) were female, and the median age was 61 years with an interquartile range (IQR) of 43.5–71.5 years. Data on vaccination status for COVID-19 was available from 84 patients, 78 (92.9%) of them were vaccinated. Number of vaccinations were documented in 77 cases. All of those had received at least 2 doses, and 26 (32.1%) had received at least 4 doses. Patient characteristics are listed in Table 1.

**Table 1:** Characteristics of the patients grouped by the primary indication for anti-CD20 mAb therapy

	All patients		Haematological malignancy		Connective tissue disease		Demyelinating disorders		Other		Statistical significance
	*N* = 98		*N* = 40		*N* = 27		*N* = 20		*N* = 11		*p-*value
Age, years	61	43.5 – 71.5	67.5	60—75	63	54.5 – 71	36.5	33 – 45	54	39 – 59	< 0.001*
Females	57/98	58.2%	18/40	45.0%	20/27	74.1%	13/20	65.0%	6/11	54.5%	0.107
BMI	26.7	23.7 – 31.2	28.0	24.6 – 30.65	28.9	24.2 – 33.6	24.8	22.7 – 27.3	28.2	25.1 – 29.0	0.55
Smoking											0.579
Never	53/94	56.4%	22/39	56.4%	15/27	55.6%	10/20	50.0%	6/8	75.0%	
Ex-smoker	34/94	36.2%	15/39	38.5%	8/27	29.6%	9/20	45.0%	2/8	25.0%	
Current smoker	7/94	7.4%	2/39	5.1%	4/27	14.8%	1/20	5.0%	0/8	-	
Anti-CD20 mAb treatment											0.212
Rituximab	94/98	95.9%	37/40	92.5%	27/27	100%	19/20	95.0%	11/11	100%	
Obinutuzumab	3/98	3.1%	3/40	7.5%	-	-	-	-	-	-	
Ocrelizumab	1/98	1.0%	-	-	-	-	1/20	5.0%	-	-	
Last dose, mg	800	500 – 1000	800	700 – 850	1000	500 – 1000	500	500 – 800	800	665 – 1000	0.014*
Days before COVID-19	85.5	38.5 – 156.0	59.5	9.5 – 161.5	113	70.5—169	94	49 – 147	188	80 – 260	0.093
Vaccinated^a^	78/84	92.9%	34/34	100%	23/23	100%	15/19	78.9%	6/8	75.0%	0.03*
Comorbidities											
Diabetes mellitus	9/98	9.2%	5/40	12.5%	3/27	11.1%	0/20	-	1/11	9.1%	0.445
Hypertension	28/98	28.6%	17/40	42.5%	8/27	29.6%	0/20	-	3/11	27.3%	0.008*
COPD	2/98	2.0%	0/40	-	1/27	3.7%	0/20	-	1/11	9.1%	0.225
Asthma	7/98	7.1%	1/40	5%	6/27	22.2%	0/20	-	0/11	-	0.005*
Sleep apnoea	11/98	11.2%	5/40	12.5%	4/27	14.8%	1/20	5.0%	1/11	9.1%	0.743
Atrial fibrillation	8/98	8.2%	4/40	10%	3/27	11.1%	0/20	-	1/11	9.1%	0.516
Coronary artery disease	7/98	7.1%	1/40	2.5%	5/27	18.5%	0/20	-	1/11	9.1%	0.043*
Kidney disease	5/98	5.1%	0/40	-	4/27	14.8%	0/20	-	1/11	9.1%	0.031*

Patient characteristics of all patients, and grouped according to primary indication for anti-CD20 treatment. Numeric data are displayed with median and quartiles (Q1 – Q3), and categorical data are displayed in numbers and percentages. Statistical significance of differences between the groups is calculated with Pearson Chi-square for categorical variables and by Kruskal Wallis 1-way ANOVA for numeric variables. BMI: body mass index, COPD: chronic obstructive pulmonary disease. ^a^77/84 (91.7%) patients had received at least 2 vaccinations (data on the number of vaccinations missing in 1 patient). * = *p-*value < 0.05

### Primary indication for anti-CD20 mAb therapy

**Fig. 1:**
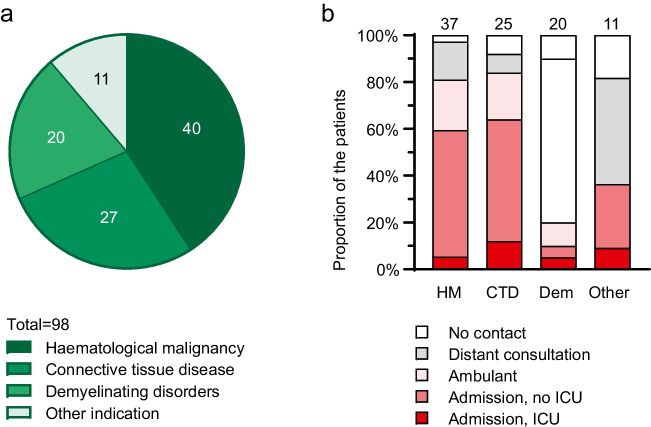
**a**) The number of patients in the groups according to primary indication for anti-CD20 mAb therapy. A total of 98 patients were on anti-CD20 mAb therapy at the time of COVID-19 diagnosis. Haematological malignancy was the primary indication for anti-CD20 mAb therapy in 40 patients, CTD in 27 patients, and demyelinating disorder in 20 patients. The group “other indication” (*n* = 11) included patients with ITP, AIHA, CVID with GL-ILD, chronic nephritic syndrome with mesangiocapillary glomerulonephritis, autoimmune hepatitis, minimal change glomerulonephritis, neuromyelitis optica spectrum disorder, and pemphigus vulgaris (listed in the Table 2). **b**) The proportion of patients in terms of the type of health care consumption. HM = haematological malignancy, CTD = connective tissue disease, Dem = demyelinating disorder, and Other = other primary indication for anti-CD20 mAb therapy. The integer numbers above the bars indicate the number of patients

We divided patients into groups according to the primary indication for anti-CD20 mAb therapy. In 40 patients (40.8%) the primary indication for anti-CD20 mAb was haematological malignancy. CTD was the primary indication in 27 patients (27.6%), demyelinating disorder in 20 patients (20.4%), and 11 patients (11.2%) had other primary indication (Fig. 1a, Table 1). The indications are further specified in Table 2.

**Table 2:** Underlying disease in the patients on anti-CD20 mAb therapy

Condition	%	Number
Haematological malignancy		*N* = 40
Diffuse large B-cell lymphoma	27.5%	11
Mantle cell lymphoma	27.5%	11
CLL / SLL	17.5%	7
Follicular lymphoma	12.5%	5
Marginal zone lymphoma	7.5%	3
Unspecified B cell lymphoma	5%	2
Waldenström macroglobulinemia	2.5%	1
Stem cell transplantation	25.0%	10
Time between SCT and COVID-19, months (median, Q1–Q3)	36.5	19 – 54
Connective tissue disease		*N* = 27
Rheumatic arthritis	48.1%	13
Granulomatosis with polyangiitis	14.8%	4
EGPA	3.7%	1
MPA	14.8%	4
Sjögren’s syndrome	11.1%	3
Systemic sclerosis	7.4%	2
CTD-associated ILD	44.4%	12
Demyelinating disease		*N* = 20
Multiple sclerosis	95.0%	19
Unspecified demyelinating disease of the central nervous system	5.0%	1
Other		*N* = 11
ITP	27.3%	3^a^
AIHA	18.2%	2
CVID with GL-ILD	18.2%	2^a^
Chronic nephritic syndrome with mesangiocapillary glomerulonephritis	9.1%	1
Autoimmune hepatitis	9.1%	1
Pemphigus vulgaris	9.1%	1
Minimal change glomerulonephritis	9.1%	1
Neuromyelitis optica spectrum disorder	9.1%	1

^a^In one patient, anti-CD20 mAb therapy was started for CVID with GL-ILD and ITP. AIHA: autoimmune haemolytic anaemia, CLL: chronic lymphocytic leukaemia, CTD: connective tissue disease, CVID: common variable immunodeficiency, EGPA: eosinophilic granulomatosis with polyangiitis, GL-ILD: granulomatous-lymphocytic interstitial lung disease, ILD: interstitial lung disease, ITP: immune thrombocytopenic purpura, MPA: microscopic polyangiitis, Q1: 1st quartile, Q3: 3rd quartile, SCT: stem cell transplantation, SLL small lymphocytic lymphoma

Text:ITP was also present in 1 patient with haematological malignancy. Rheumatic arthritis was also present in 2 patients with haematological malignancy and in 1 patient with multiple sclerosis. SLE was not the primary indication for anti-CD20 in any of the patients but was a comorbidity in 2 patients with other indication (CVID with GL-ILD). Sjögren’s syndrome was a comorbidity in 1 patient with haematological malignancy

These groups differed from each other in age (*p* < 0.001) and in the last dose of anti-CD20 mAb (*p* = 0.014) but not in the time between the most recent administration and onset of symptoms (*p* = 0.093). Furthermore, patients with demyelinating disorder had less comorbidities and comedications than patients in other groups. One patient with MS was 14 weeks pregnant. Recent comedications that strongly affect immunity in study patients are listed in Supplementary Table 1. Twenty-six of 98 patients (26.5%) used systemic corticosteroids on a regular basis but only in 4 patients the daily dose was more than 10 mg prednisone equivalent. Comorbidities are presented in Table 1 and the most relevant comedications in Supplementary Table 1.

### Clinical course and treatment characteristics of COVID-19

Typical clinical findings upon presentation were overall mild-to-moderate with normotension (median blood pressure 127/73 mmHg), mild tachycardia (median pulse 92/min), mild fever (median body temperature 38.0°C), and a median peripheral oxygen saturation of 96% on ambient air. Most laboratory parameters were within the normal range while the median values for infection biomarkers were at most moderately elevated. However, laboratory parameters were not available in all patients and are likely skewed towards patients with severe infection. The baseline clinical parameters are presented in Supplementary Table 2.

Of 98 patients, 5 patients (5%) were admitted to hospital for another indication than COVID-19 and 44 patients (47%) were hospitalized for COVID-19, as depicted in Table 3. Thirty-two patients (33%) received respiratory support while systemic corticosteroids were administered in 31 cases (32%). Patients with demyelinating disorder as the primary indication for anti-CD20 mAb were less likely to be admitted and required less invasive treatment, but they also displayed a favourable risk profile regarding age, comorbidities, and co-medications (Fig. 1b, Tables 1 and 3, and Supplementary Table 1).Antivirals (remdesivir and/or nirmatrelvir-ritonavir) were administered within the first week of symptoms in 17/30 patients (56.7%) (1 missing value) while tixagevimab-cilgavimab was administered only in 2/9 patients (22.2%) within the first week of symptoms. One patient received remdesivir for eight days but all the others at the maximum of five days. Nirmatrelvir-ritonavir was used as a five-day course in all cases.

**Table 3:** COVID-19 treatment characteristics of all patients and grouped according to primary indication for anti-CD20 mAb therapy

	All patients		Haematological malignancy		Connective tissue disease		Demyelinating disorders		Other		Statistical significance
	*N* = 98		*N* = 40		*N* = 27		*N* = 20		*N* = 11		*p-*value
Health care consumption^a^											< 0.001*
No consultation	7/93	7.5%	1/37	2.7%	2/25	8.0%	2/20	10.0%	2/11	18.2%	
Only distant consultation	27/93	29.0%	6/37	16.2%	2/25	8.0%	14/20	70.0%	5/11	45.5%	
Ambulant (ED or OPD)	15/93	16.1%	8/37	21.6%	5/25	20.0%	2/20	10.0%	0/11	-	
Hospitalized for COVID-19	44/93	47.3%	22/37	59.5%	16/25	64.0%	2/20	10.0%	4/11	36.4%	
Requiring ICU	7/93	7.5%	2/37	5.4%	3/25	12.0%	1/20	5.0%	1/11	9.1%	
Hospitalized for other indication	5/98	5.1%	3/40	7.5%	2/27	7.4%	0/20	-	0/11	-	
Maximal respiratory support											0.102
No respiratory support	66/98	67.3%	25/40	62.5%	15/27	55.6%	19/20	95.0%	7/11	63.6%	
Low-flow supplemental oxygen	19/98	19.4%	10/40	25.0%	6/27	22.2%	1/20	5.0%	2/11	18.2%	
HFNC	3/98	3.1%	2/40	5.0%	0/27	-	0/20	-	1/11	9.1%	
NIV	7/98	7.1%	2/40	5.0%	5/27	18.5%	0/20	-	0/11	-	
MV	3/98	3.1%	1/40	2.5%	1/27	3.7%	0/20	-	1/11	9.1%	
COVID-19 medical treatment											
Systemic corticosteroids	31/98	31.6%	14/40	35.0%	13/27	48.1%	1/20	5.0%	3/11	27.3%	0.016*
Tocilizumab	4/98	4.1%	2/40	5.0%	1/27	3.7%	0/20	-	1/11	9.1%	0.648
Remdesivir	12/98	12.2%	7/40	17.5%	4/27	14.8%	0/20	-	1/11	9.1%	0.252
Nirmatrelvir-ritonavir	18/98	18.4%	10/40	25.0%	5/27	18.5%	3/20	15.0%	0/11	-	0.284
Tixagevimab-cilgavimab	9/98	9.2%	5/40	12.5%	4/27	14.8%	0/20	-	0/11	-	0.196
LMWH	34/98	34.7%	17/40	42.5%	13/27	48.1%	1/20	5.0%	3/11	27.3%	0.010*
COVID-19 related death	13/98	13.3%	7/40	17.5%	4/27	14.8%	0/20	-	2/11	18.2%	0.265

Numeric data are displayed with median and quartiles (Q1 – Q3), and categorical data are displayed in numbers and percentages. Statistical significance of differences between the groups is calculated with Pearson Chi-square for categorical variables and by Kruskal Wallis 1-way ANOVA for numeric variables. ED: emergency department, HFNC: high-flow nasal cannula, ICU: intensive care unit, LMWH: low-molecular-weight heparin, MV: mechanical ventilation, NIV: non-invasive ventilation, OPD: outpatient department. ^a^Patients admitted for other indication than COVID-19 are excluded. * = *p-*value < 0.05

For hospitalized patients, low-molecular-weight heparin (LMWH) was administered as a higher-than-normal prophylactic dose (typically 0.5 mg/kg enoxaparin twice daily) except if there were risk factors for bleeding. Ten patients did not receive LMWH during hospitalization. Six of them had other anticoagulants as regular medication which was not changed into LMWH. Two patients had severe thrombocytopenia as a contraindication for anticoagulation, and for two patients the reason for not giving LMWH was unclear. Treatment data is presented in Table 3.

### COVID-19 related mortality

**Fig. 2:**
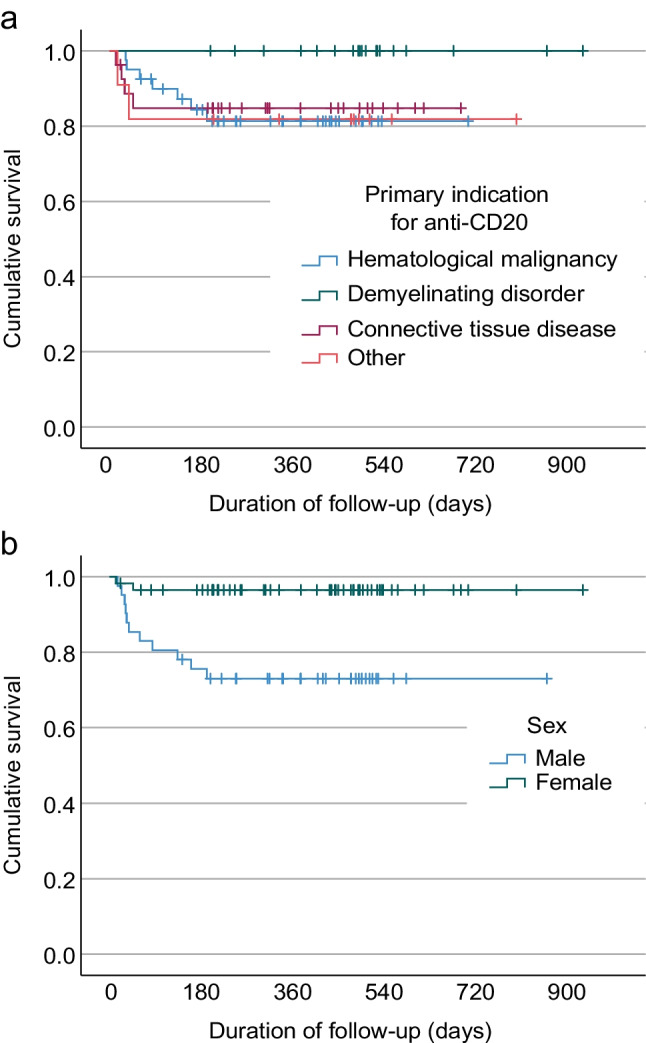
Survival of patients in the groups according to primary indication for anti-CD20 mAb therapy (**a**) and gender (**b**)

Of 98 patients on anti-CD20 mAb therapy, 13 (13.3%) died because of COVID-19 (Table 4 and Fig. 2). The median age of the patients who died was 69 years (IQR 55–77.5 years), and 11/13 (84.6%) were male (*p* < 0.001). In binary regression, unadjusted odds ratio was 10.1 for male gender (95% confidence interval 2.1–48.5, *p* = 0.004) and 1.052 per year for age (1.005–1.102, *p* = 0.03). Among deaths, haematological malignancy was the primary indication for anti-CD20 mAb therapy in 7/13 patients (all males), CTD in 4/13 patients (2 males and 2 females), demyelinating disorder in 0/13 patients, and other primary indication for anti-CD20 mAb therapy in 2/13 patients (both males) (Supplementary Fig. 1), while the absence of events among patients with a demyelinating disorder hampered logistic regression and multivariate analysis. Most of those who died had co-morbidities such as primary hypertension (7/13; 53.8%), atrial fibrillation (3/13; 23.1%), type 2 diabetes (2/13; 15.4%), coronary artery disease (2/13; 15.4%), asthma (1/13; 7.7%), or chronic obstructive pulmonary disease (1/13; 7.7%), and 12/13 had been vaccinated at least twice while the vaccination status was missing in one case.

**Table 4:** Baseline characteristics of the patients who died and survived, and the use of COVID-19 medication during the hospitalization

	Death^a^ (*n* = 13)		Survived (*n* = 85)		*p-*value
Females	2/13	15.4%	55/85	64.7%	< 0.001*
Age, years	69	55─77.5	58	42─69	0.025*
BMI (kg/m^2^)	25.8	24.6─31.0	27.7	23.8─30.9	0.921
Days between anti-CD20 mAb and symptoms	82	39─146	100	39─177	0.543
Duration of symptoms at time of arrival (days)	18	5─22	6	2─16	0.124
Dose of anti-CD20 mAb (mg)	800	700─1000	700	500─1000	0.132
Charlson comorbidity index (points)	4	2.5─5.5	3	0.5─4	0.033*
COVID-19 medication					
Systemic corticosteroids	11	84.6%	20	23.5%	< 0.001*
Tocilizumab	2	15.4%	2	2.4%	0.027*
Remdesivir	6	46.2%	6	7.1%	< 0.001*
Nirmatrelvir-ritonavir	1	7.7%	17	20.0%	0.286
Tixagevimab-cilgavimab	2	15.4%	7	8.2%	0.406
LMWH	10	76.9%	24	28.2%	< 0.001*

Numeric data are displayed with median and quartiles, and categorical data are displayed in numbers and percentages. BMI: body mass index, LMWH: low-molecular-weight heparin. ^a^Among those who died due to COVID-19, the indication for anti-CD20 mAb treatment was haematological malignancy in 7 patients (all males) and CTD in 4 patients (2 males and 2 females). Two male patients who died had other indication for anti-CD20 mAb. * = *p-*value < 0.05

Among fatal COVID-19 cases, the median time from the most recent administration of anti-CD20 mAb therapy to the onset of symptoms was 82 days (IQR 39─146 days), and from the onset of symptoms to death 18 days (IQR 5─22 days).

### Features of prolonged pneumonia

In 33 of 94 (35.1%) patients alive by day 30, respiratory symptoms and/or fever were still present after 30 days from the onset of COVID-19. This was still the case in 21/89 (23.6%) patients after 60 days, and in 15/85 (17.6%) patients after 90 days (Table 5). Patients reporting prolonged respiratory symptoms and/or fever after COVID-19 were almost exclusively those with haematological malignancy or CTD.

**Table 5:** Features of prolonged pneumonia in all patients and grouped according to primary indication for anti-CD20 mAb therapy

	All patients		Haematological malignancy		Connective tissue disease		Demyelinating disorder		Other		Statistical significance
	*N* = 98		*N* = 40		*N* = 27		*N* = 20		*N* = 11		*p-*value
30 days from start of symptoms (*N* = 94)											
Respiratory symptoms	30/94	31.9%	18/40	45.0%	11/24	45.8%	0/20	-	1/10	10.0%	< 0.001*
Fever	26/94	27.7%	18/40	45.0%	7/24	29.2%	0/20	-	1/10	10.0%	0.002
Radiologic findings^a^	30/39	76.9%	18/24	75.0%	10/12	83.3%	1/1		1/2		
Positive PCR^b^	21/28	75.0%	14/16	87.5%	5/9	55.6%	0/0		2/3		
60 days from start of symptoms (*N* = 89)											
Respiratory symptoms	19/89	21.3%	12/38	31.6%	7/22	31.8%	0/20	-	0/9	-	0.009*
Fever	16/89	18.0%	11/38	28.9%	5/22	22.7%	0/20		0/9		0.020*
Radiologic findings^a^	19/32	59.4%	12/21	57.1%	6/9	66.7%	1/1		0/1		
Positive PCR^b^	15/23	65.2%	10/14	71.4%	4/7	57.1%	0/0		1/2		
90 days from start of symptoms (*N* = 85)											
Respiratory symptoms	15/85	17.6%	9/34	26.5%	6/22	27.3%	0/20	-	0/9	-	0.024*
Fever	9/85	10.6%	5/34	14.7%	4/22	18.2%	0/20	-	0/9	-	0.146
Radiologic findings^a^	17/33	51.5%	9/19	47.4%	7/11	63.6%	1/2		0/1		
Positive PCR^b^	9/21	42.9%	5/10	50.0%	4/8	50.0%	0/1		0/2		

Numeric data are displayed with median and quartiles (Q1 – Q3), and categorical data are displayed in numbers and percentages. Statistical significance of differences between the groups is calculated with Pearson Chi-square for categorical variables and by Kruskal Wallis 1-way ANOVA for numeric variables. PCR: polymerase chain reaction. ^a^Only patients with radiologic investigation included, ^b^Only patients with PCR (re-)tested. * = *p-*value < 0.05

**Fig. 3:**
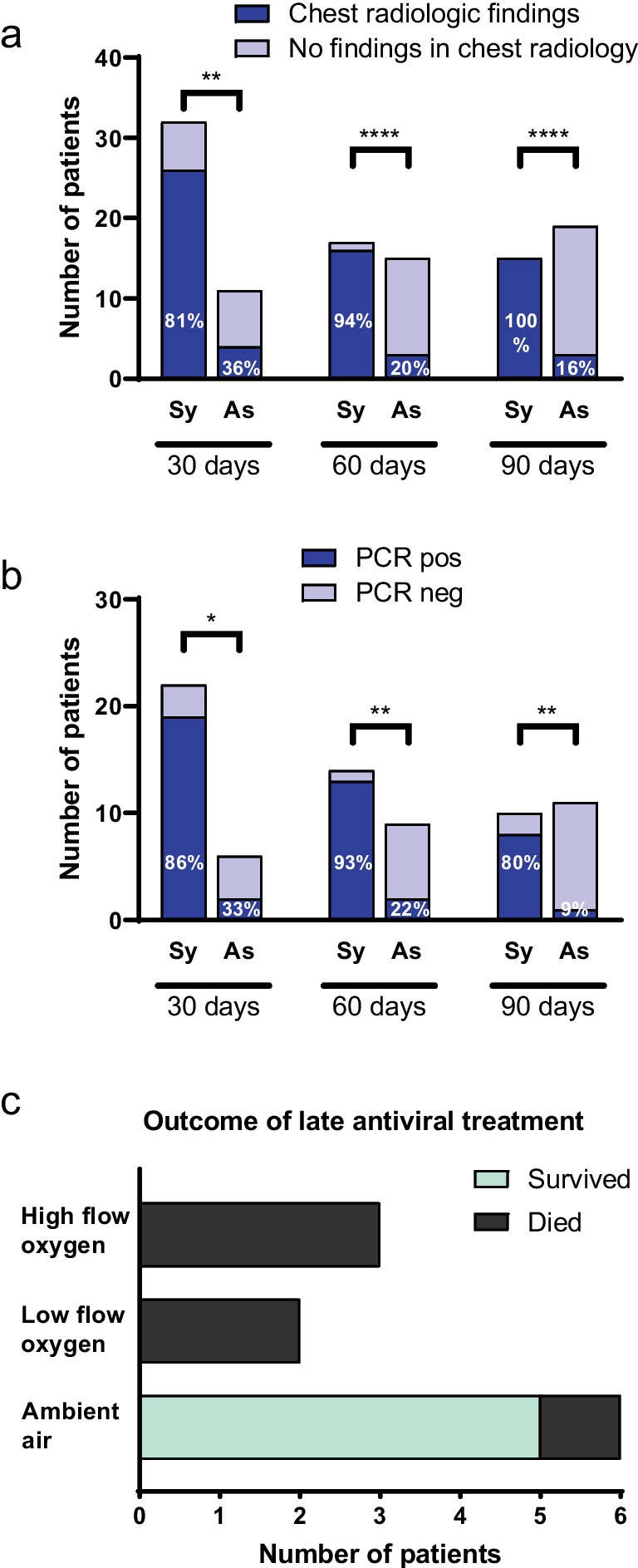
Features consistent with prolonged viral pneumonia. a-b) The proportion of symptomatic (Sy) and asymptomatic (As) patients with and without radiologic findings (**a**) in chest CT and (**b**) SARS-CoV-2 RT-PCR test results after 30, 60, and 90 days from the start of the COVID-19-related symptoms. Statistical significance of differences between the groups was calculated with Pearson Chi-square test. *P-*value < 0.05 is considered statistically significant. * *p* < 0.05; **p < 0.01; ****p < 0.0001. c) Outcome of late antiviral treatment. Eleven patients received an antiviral after more than 1 week of symptom onset. The clinical outcome was highly dependent on the level of respiratory support at the time of initiation of antiviral medication (*p* = 0.0057)

Chest computed tomography (CT) was performed for around a third of the patients after 30, 60, and 90 days after the COVID-19 onset. Even after 90 days, symptomatic patients had COVID-19-related radiologic findings, such as ground glass opacities, whereas most of the asymptomatic patients did not have, and the difference between these groups was statistically significant: 100% vs. 16%, OR infinite (95% CI 11.9–infinity, *p* < 0.0001) (Fig. 3a).

In a proportion of patients, nasopharyngeal (NP) samples were analysed by SARS-CoV-2 RT-PCR after 30, 60, and 90 days of the COVID-19 onset. After 30 days, 21/28 (75.0%) were positive for SARS-CoV-2, after 60 days 15/23 (65.2%), and after 90 days 9/21 (42.9%) (Table 5). Patients with prolonged symptoms were more likely to have prolonged PCR-positivity than patients with no prolonged symptoms reported: 86% vs. 33% after 30 days, OR 12.7 (95%CI 1.6–78.6, *p* = 0.0207); 93% vs. 22% after 60 days, OR 45.5 (95%CI 4.0–535.0, *p* = 0.0010); and 80% vs. 9% after 90 days, OR 40 (95%CI 3.5–472.0, *p* = 0.0019) (Fig. 3b). The reasons for radiological evaluation and RT-PCR were not gathered for this study.

### Late antiviral treatment

Eleven patients received an antiviral treatment after more than 1 week of symptom onset. The clinical outcome was highly dependent on the level of respiratory support at the time of initiation of antiviral medication (*p* = 0.0057). None of the 5 patients with respiratory support survived (3 on HFNC and 2 on low flow oxygen), whereas 5/6 patients on ambient air survived (Fig. 3c). Rapid complete resolution of symptoms within one week was observed in 3 patients on ambient air after 23, 98, and 282 days of symptoms, respectively. In two of those patients (with 98 and 282 days of symptoms), radiologic findings consistent with organizing pneumonia also rapidly resolved after antiviral medication. In one of the patients on low flow oxygen therapy, an initial complete resolution of symptoms was followed by a relapse of symptoms after one week and eventually death. In one patient, the researchers noted clinical findings consistent with prolonged viral pneumonia from the patient files when performing this study. In this case, the treating physician was notified. SARS-CoV-2 was detected from NP sample as well as from bronchoalveolar lavage (BAL) fluid sample, and sequencing confirmed infection with Omicron BA.2, which was the dominant strain at the time of symptom onset 1.5 years earlier. A course of antiviral medication (nirmatrelvir-ritonavir) was started after 550 days of symptom onset, followed by tixagevimab-cilgavimab injection at day 555. At the time of writing this manuscript, the treatment outcome was not yet assessable. During the review process, it was documented that all symptoms resolved by the 4th day after tixagevimab-cilgavimab. Three months after treatment, radiologic manifestations were resolved, and RT-PCR was negative. Figure 3c was not adapted as these findings were obtained only after the manuscript was submitted.

## Discussion

The clinical observation in our hospital that patients on anti-CD20 mAb therapy seemed prone to contract severe or prolonged COVID-19, prompted us to explore the clinical picture of COVID-19 in this unique group of immunosuppressed patients. Here, we report severe disease and a high mortality in patients on anti-CD20 mAb therapy. Furthermore, a substantial proportion of patients had prolonged symptoms and clinical findings consistent with subacute or chronic viral pneumonia.

In our cohort, almost half of the patients were hospitalized due to COVID-19 within the study period, and 7.5% required intensive care. This is in line with previous studies, which indicated that the use of B-cell depleting anti-CD20 mAbs are associated with higher occurrence of severe, sometimes fatal, COVID-19 and prolonged hospital stay in patients with haematologic malignancies or rheumatologic diseases [22–24]. Patients receiving anti-CD20 mAbs may be immunosuppressed due to the primary disease as well as due to the treatment and might thus be vulnerable to contract more severe and prolonged SARS-CoV-2 infection [7]. Other previously reported predictors for severe COVID-19 including mortality in immunosuppressed patients are male sex, advanced age, chronic use of systemic corticosteroids, active haematologic malignancy with lymphopenia, and other comorbidities [25–27]. As far as we are aware of, we are the first to evaluate the frequency of this prolonged phenotype in a larger study population of patients on anti-CD20 mAb therapy for different indications.

In our cohort, immunosuppression may not only be the consequence of anti-CD20 mAb therapy, but may also be caused by the primary disease, comorbidities, and comedications. Clinical outcome among patients with demyelinating disorder was clearly better than in the other groups. Patients in this group were also substantially younger and had less comorbidities and comedications. Multivariate analysis by logistic regression could not take this group into consideration due to the absence of events (mortality) in this group and was therefore inconclusive. Any multivariate analysis was further hampered by the clear differences between the patient groups in a number of factors in combination with the relatively small number of subjects. Thus, from our data, we cannot conclude whether poor outcome in our cohort is primarily due to anti-CD20 mAb therapy or to other factors. A systematic review performed in 2021 found that MS was not associated with increased mortality in COVID-19 [28]. However, a Swedish register study performed around the same time showed that rituximab was associated with increased risk of hospitalization in MS patients [29]. Furthermore, a large study of COVID-19 severity outcomes in people with MS based on clinical data gathered from 27 countries concluded that the use of anti-CD20 mAb therapy is associated with more severe COVID-19 in terms of hospitalization, ICU/artificial ventilation, and death [30].

The vast majority of the patients who died from COVID-19 were male. The mortality was significantly higher among men than women. Despite the similar numbers of COVID-19 cases in men and women, epidemiological data from several countries show the sex imbalance with men being at a higher risk of more severe SARS-CoV-2 infection and death across the age-groups [31]. Sex‐based differences in immune responses have been reported for adults and children, resulting from the influence of X chromosome and sex hormones on the immune system [32]. Numerous other biological and social factors, such as comorbidities, tobacco smoking, or propensity to seek health care, may also have a role in the observed sex disparity in COVID-19 [31].

In immunocompetent individuals, symptoms typically abate within 5─7 days after COVID-19 onset, and nearly all achieve viral clearance by 28 days [33]. Among our cohort of patients, in a substantial proportion of patients in whom chest CT and/or NP sample for SARS-CoV-2 RT-PCR testing was taken beyond the acute phase of infection, findings were consistent with prolonged pneumonia. Most symptomatic patients that were investigated had persistent radiologic findings (81─100%) and positive SARS-CoV-2 RT-PCR result (80─93%) in comparison with asymptomatic patients. Based on qualitative result of RT-PCR, we cannot be completely sure whether the positive PCR results represent viable viruses and an ongoing viral replication in our patients. However, considering the low percentage of PCR positive asymptomatic patients and the significant difference between symptomatic and asymptomatic patients in PCR positivity as well as in the frequency of radiologic findings, we assume that the positive PCR results do not represent non-infectious viral particles as a remnant of the previous acute infection but rather reflect an active, persistent viral shedding. In addition, the observation of rapid complete resolution of symptoms within one week after administration of antiviral medication in 3 patients after 23, 98, and 282 days of symptoms also supports the concept of prolonged viral pneumonia in these patients. In previous reports, prolonged viral shedding including with viable viruses, has been reported to last up to months in patients with different forms of immunosuppression [11, 15, 23, 34–40]. In addition, a number of case reports have described prolonged viral replication over months after the primary infection, and persistent viral pneumonia in immunocompromised patients, including those with B-cell depleting therapies [12, 16, 18, 19, 34, 41–46]. It is important to differentiate possible chronic viral pneumonia from other causes of prolonged symptoms after acute COVID-19 as this may be treatable with antiviral therapy.

Among patients with prolonged symptoms, the proportion displaying SARS-CoV-2 RT-PCR positivity was considerably high, especially when taken into consideration that all the patients were tested positive from NP swabs, and not from lower respiratory tract specimens. Based on previous reports on immunocompromised patients, clinical relapse may occur despite early viral clearance from nasopharynx, in which case viral replication might still be detected in low respiratory tract samples [11–13]. Thus, bronchoscopy with BAL is recommended especially in immunocompromised patients with the high clinical suspicion for COVID-19 and negative NP sample [7].

Numerous studies on patients with haematological malignancies or autoimmune diseases receiving B-cell depleting therapies have reported the paramount role of antibody-mediated immune response on achieving viral clearance in patients with prolonged viral shedding [47, 48]. However, also reports of patients recovering from COVID-19 without developing neutralizing antibodies against SARS-CoV-2 have been published, demonstrating that viral clearance and clinical recovery may happen despite impaired B cell response [49]. Recent immunophenotyping findings in patients with cancer have revealed that well-functioning CD8 + T cell response might be critical in surviving acute SARS-CoV-2 infection whereas B cells and CD4 + T cells are needed in achieving the ultimate viral clearance and clinical recovery [50]. Unfortunately, the neutralizing antibody levels against SARS-CoV-2, T cell subsets and B cell counts were not systematically taken in our study patients.

Our study has several limitations. First, due to the retrospective nature of the study and with absence of management guidelines for COVID-19 in immunosuppressed patients, there was no consistent management of cases. Therefore, the existence of prolonged symptoms and other signs of prolonged disease may have been missed in a substantial proportion of patients. In addition, anamnestic and clinical findings may have been under-reported for those who did not present at the hospital. Thus, the percentages of prolonged PCR positivity and pathological findings in chest CT may be flawed. Cycle threshold values and viral genome sequencing data were only incidentally requested. Second, mild infections may have been missed especially in the later phase of the pandemic as the testing policy changed in early 2022 which might cause a bias. Third, since no control group was included, it is difficult to evaluate the surplus risk related to anti-CD20 mAb therapy in addition to underlying conditions causing severe or prolonged viral pneumonia. Fourth, multivariate analysis was hampered by the low number of patients and the absence of COVID-19 related mortality among patients receiving anti-CD20 mAb for demyelinating disorders.

In conclusion, our study indicates that prolonged viral pneumonia should be considered in COVID-19 patients on anti-CD20 mAb therapy. These patients could benefit from routine treatment with antivirals in the early phase of infection. Furthermore, our study provides rationale for retesting patients for SARS-CoV-2 on NP and/or BAL fluid samples, and consider antiviral medication followed by antibody therapy in immunosuppressed patients with findings suggestive of prolonged viral SARS-CoV-2 pneumonia. Future studies could address the possibility of prolonged pneumonia related to other viral infections in immunocompromised patients. Furthermore, we conclude that four years after the identification of SARS-CoV-2, immunocompromised patients are still poorly addressed in COVID-19 guidelines.

### Supplementary Information

Below is the link to the electronic supplementary material.

Supplementary file1 (EPS 1455 KB) Fig. 1 Survival of patients in the groups according to primary indication for anti-CD20 mAb therapy in males (a) and females (b)

Supplementary file2 (DOCX 17 KB) Table 1 Co-medications in the patient groups according to primary indication for anti-CD20 mAb therapy
Table 2 Clinical parameters at baseline of the patients who presented at the emergency department and/or were admitted to the hospital

## Data Availability

Anonymized data is available from the corresponding author on reasonable request.
