# Supplementary material for: Prolonged viral pneumonia and high mortality in COVID-19 patients on anti-CD20 monoclonal antibody therapy
Source: Eur J Clin Microbiol Infect Dis. 2024 Feb 15;43(4):723–34. doi: 10.1007/s10096-024-04776-0 (PMC10965662; doi:10.1007/s10096-024-04776-0)
Supplement: Supplementary file 2 — Supplementary file2 (DOCX 17 KB) Table 1 Co-medications in the patient groups according to primary indication for anti-CD20 mAb therapy Table 2 Clinical parameters at baseline of the patients who presented at the emergency department and/or were admitted to the hospital [file 10096_2024_4776_MOESM2_ESM.docx]

**Supplementary table 1:** Co-medications in the patient groups according to primary indication for anti-CD20 mAb therapy.

| Condition | Medications | % | n/N |
| --- | --- | --- | --- |
| Haematological malignancy | Pegylated filgastrim | 35.0% | 14 /40 |
|  | Cyclophosphamide | 27.5% | 11 /40 |
|  | Doxorubin | 22.5% | 9/40 |
|  | Vincristin | 22.5% | 9/40 |
|  | Bendamustine | 20.0% | 8/40 |
|  | Etoposide | 15.0% | 6/40 |
|  | Systemic corticosteroids (daily) | 10.0% | 4/40 |
|  | Cytaribin | 10.0% | 4/40 |
|  | Venetoclax | 7.5% | 3/40 |
|  | Mycophenolate | 7.5% | 3/40 |
|  | Methotrexate | 5.0% | 2/40 |
|  | Immunoglobulins | 2.5% | 1/40 |
| Connective tissue disease | Systemic corticosteroids | 55.6% | 15/27 |
|  | Hydroxychloroquine | 25.9% | 7/27 |
|  | Methotrexate | 22.2% | 6/27 |
|  | Sulfasalazin | 18.55 | 5/27 |
|  | Mycophenolate | 11.1% | 3/27 |
|  | Azathioprine | 7.4% | 2/27 |
|  | Cyclophosphamide | 3.7% | 1/27 |
| Demyelinating disorder | Systemic corticosteroids | 5% | 1/20 |
|  | Hydroxychloroquine | 5% | 1/20 |
| Other indications | Systemic corticosteroids | 54.5% | 6/11 |
|  | Mycophenolate | 27.3% | 3/11 |
|  | Immunoglobulin treatment | 18.2% | 2/11 |
|  | Hydroxychloroquine | 18.2% | 2/11 |
|  | Cyclosporin | 9.1% | 1/11 |

Recent comedications strongly affecting immunity in groups according to primary indication for anti-CD20 therapy.

**Supplementary table 2:** Clinical parameters at baseline of the patients who presented at the emergency department and/or were admitted to the hospital.

| Clinical parameter | Median | Q1 – Q3 | Number |
| --- | --- | --- | --- |
| Physical examination |  |  |  |
| Systolic blood pressure (mmHg) | 127 | 120 – 138 | 52 |
| Diastolic blood pressure (mmHg) | 73 | 64.5 - 83 | 52 |
| Pulse (b/min) | 92 | 77 – 101 | 51 |
| Native oxygen saturation (%) | 96 | 93 – 98 | 53 |
| Temperature (°C) | 38.0 | 37.2 – 38.7 | 50 |
| Laboratory findings |  |  |  |
| C-reactive protein (mg/L) | 41 | 20 – 69.5 | 55 |
| ESR (mm/h) | 49 | 35.5 – 82.5 | 31 |
| Procalcitonine (µg/L) | 0.13 | 0.075 – 0.245 | 32 |
| Haemoglobin (g/L) | 118 | 102 – 135 | 57 |
| Blood leukocytes (x10^9^/L) | 5.1 | 3.6 – 7.6 | 57 |
| Blood lymphocytes (x10^9^/L) | 0.49 | 0.27 – 0.78 | 38 |
| Ferritin (µg/L) | 1131 | 468 – 1739 | 30 |
| Interleukin-6 (ng/L) | 177 | 117 – 487 | 31 |
| D-dimer (mg/L) | 0.7 | 0.5 – 1.7 | 29 |
| MxA (µg/L) | >800 | 738 – >800 | 32 |
| Lactate dehydrogenase (IU/L) | 309 | 237 – 397 | 33 |
| Creatinine (µmol/L) | 77.0 | 61.5 – 94 | 55 |
| ALT (IU/L) | 22 | 16 – 35.5 | 43 |
| Days since start symptoms | 7 | 2.5 – 18 | 59 |

ALT: alanine aminotransferase, ESR: erythrocytes sedimentation rate, MxA: myxovirus resistance protein 1.
